# Fixation durations on familiar items are longer due to attenuation of exploration

**DOI:** 10.1186/s41235-024-00602-5

**Published:** 2024-11-14

**Authors:** Tal Nahari, Eran Eldar, Yoni Pertzov

**Affiliations:** 1 Cognitive and Brain Sciences, The Hebrew University of Jerusalem, Jerusalem, Israel; 2 Affective Brain Lab, Department of Experimental Psychology, University College London, London, UK; 3 Max Planck UCL Centre for Computational Psychiatry and Ageing Research, University College London, London, UK; 4 Psychology Department, The Hebrew University of Jerusalem, Jerusalem, Israel

## Abstract

Previous studies have shown that fixations on familiar stimuli tend to be longer than on unfamiliar stimuli, theorized to be a result of retrieval of information from memory. We hypothesize that extended fixations are due to a lesser need to explore an already familiar stimulus. Participant's gaze was tracked as they tried to encode or retrieve a familiar face displayed either alone or alongside other unfamiliar faces. Regardless of the memory task (encoding\retrieval), longer fixation durations were observed when a single familiar face was presented alone, and not when presented among unfamiliar ones. Thus, fixations were not prolonged when it was possible to explore other, unfamiliar stimuli. We conclude that prolonged fixations on familiar stimuli reflect a lesser need to explore an already familiar percept. The results underscore how memory representations influence active sensing, yielding fresh insights into efficient deployment of attention resources. We conclude that fixation durations could be used in applied memory detection tests, preferably together with other measures and when the familiar stimulus is presented alone.

**Supplementary Information:**

The online version contains supplementary material available at 10.1186/s41235-024-00602-5.

## Significance statement

This study addresses a critical question in cognitive psychology and visual perception: How do our eyes behave when we encounter familiar versus unfamiliar faces, and what does this tell us about how we process information? Previous research has shown that people tend to fixate at familiar faces longer than unfamiliar ones. This has been thought to occur because recognizing familiar faces triggers our memory. We propose a different explanation: people have a lesser need to explore an already familiar face. To test this, we tracked how long participants fixated at familiar and unfamiliar faces under different conditions—either looking on a single face or multiple faces at once. Our findings reveal that when a familiar face was viewed alone, participants fixated at it for a longer time. However, when the familiar face was displayed alongside unfamiliar faces, this prolonged fixation disappeared, suggesting that the duration of gaze is linked to the opportunity to explore. These findings provide valuable insights into how our memory influences how we look. Understanding this interaction can lead to more effective strategies for memory detection in real-world settings, such as testing for concealed memories in crime investigation.

## Introduction

The quest for understanding how memories are reflected in behavior has been the target of many scholars. It has been addressed not only as a theoretical question about the structure of memory and the bodily manifestations of it, but also as an applicative tool—valuable both for detection of conscious awareness of individuals in different awareness states (Owen & Coleman, 2008); and in forensic scenarios in which crime-related knowledge is concealed (Verschuere et al., 2011).

Recent studies have started to examine eye tracking as a tool for detection of concealed information showing its’ promise to the field, relying on the way gaze behavior is affected by memory (Lancry-Dayan et al., 2023). These approaches utilized different tasks, such as working memory tasks, and yielded larger effect sizes than other traditional psychophysiological measures (Lancry-Dayan et al., 2018; Millen et al., 2020; Peth et al., 2013). Gaze is especially easy to collect relative to other high-performance techniques like EEG (Lancry-Dayan et al., 2023), and seem to be less prone to countermeasures (Lancry-Dayan et al., 2021; Millen & Hancock, 2019). However, the exact mechanism for the way familiarity affects eye measures is still unclear.

In parallel, other research has focused on the determinants of fixations (Salthouse & Ellis, 1980; Tatler et al., 2017). It is especially interesting to consider fixations as a marker of information exploitation, emerging naturally in behavior unlike other measures of eye tracking such as viewing duration (Hills et al., 2015). Fixation duration was found to be related both to planning of the next eye movement (Tatler et al., 2017), and stimulus properties and processing (Salthouse & Ellis, 1980). The contribution of the number of fixations to memory performance has been shown before in the context of working memory (Pertzov, 2009). Furthermore, it has been suggested that a unique marker of recognition should be evident in low-level gaze behaviors, and would disclose the information of past encounters even in basic eye movements, i.e., the fixation durations (Peth, 2013; Ryan, 2007). Moreover, several studies found this unique marker of memory recognition specifically in the second fixation duration (Schwedes & Wentura, 2012, 2016; Van Belle, 2010), claiming that it is caused by retrieval from memory (Schwedes & Wentura, 2012), or due to holistic processing of familiar stimuli (Millen et al., 2020) and therefore could be used as an applicative tool for memory detection.

Several studies have showed that observers tend to employ longer fixations (the relatively stable periods between eye movements) on familiar stimuli (Lancry-Dayan et al., 2018; Millen & Hancock, 2019; Nahari et al., 2019; Peth et al., 2013; Ryan et al., 2007; Schwedes & Wentura, 2019), and attributed this phenomena to retrieval of information from memory (Schwedes & Wentura, 2016, 2019) or holistic processing of familiar stimuli (Millen et al., 2020). Interestingly, studies were inconsistent in their findings: whether it was the first fixation that was longer (Ryan et al., 2007); the second fixation (Ryan et al., 2007a; Schwedes & Wentura, 2012, 2016); or the average fixation duration (Millen & Hancock, 2019; Peth et al., 2013). Some studies did not find either of the fixation durations to be significant (Millen et al., 2020). Thus, a lingering question remained regarding the conditions in which fixation durations become longer, and the mechanism behind it.

Longer fixations on familiar stimuli may appear somewhat counter-intuitive, as one might anticipate that we should invest more time fixating on unfamiliar, rather than on familiar stimuli. This assumption makes intuitive sense as novel information should demand more time to process, unlike familiar stimuli which already have a representation in mind (Jackson & Raymond, 2008; Ramon & Gobbini, 2018).

Therefore, it is interesting to examine what is the mechanism driving the longer fixation durations effect, which is what the current study is sought to explore. We consider three different hypotheses behind longer fixations on familiar stimuli: based on previous hypothesis, it could be that an internal retrieval from memory takes time, and therefore retrieval during fixation on familiar stimuli delays the next saccade. We further wished to clarify: if indeed fixation durations are longer due to retrieval, is this retrieval automatic (i.e., happens whenever a memory representation appears), or only when instructed to retrieve items from memory? Alternatively, we raised another hypothesis: longer fixations may be an indication of a lesser need to execute the next saccade and therefore to reflect an attenuation of visual exploration.

**Fig. 1:**
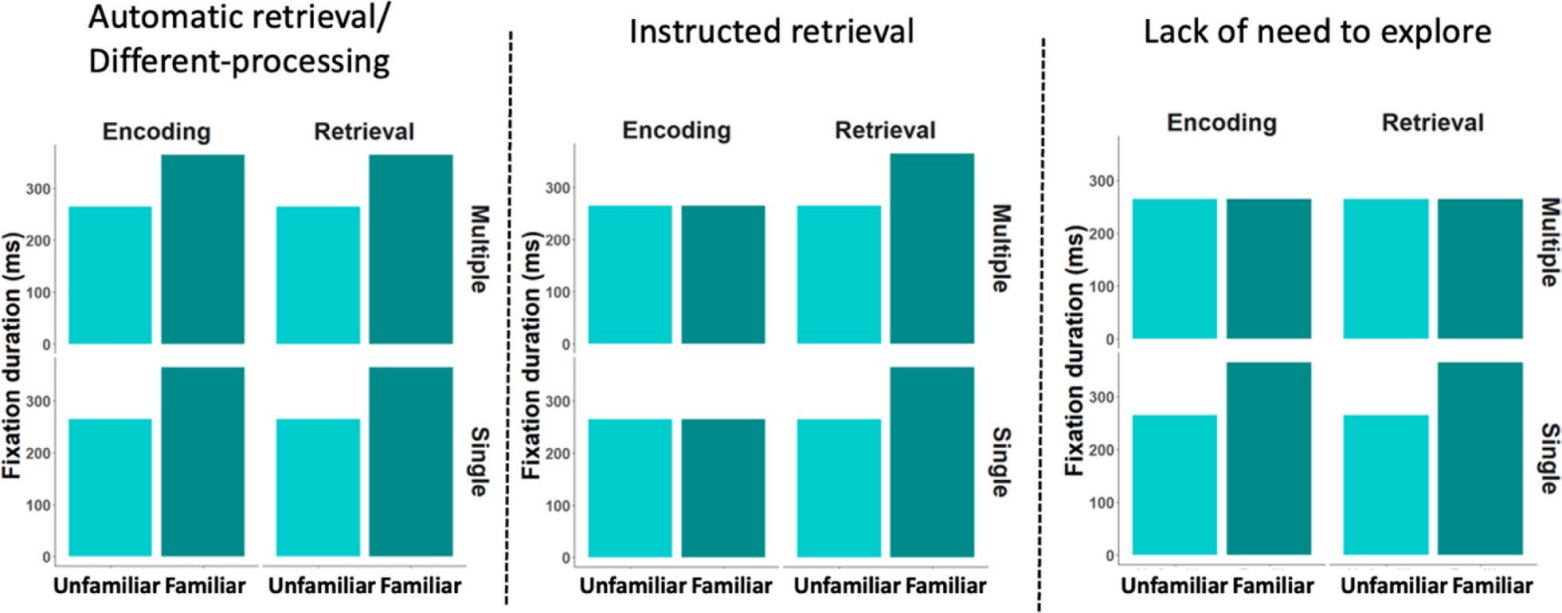
Predicted results of the three hypotheses regarding the mechanism behind the increase in fixation durations on familiar stimuli. In the case of automatic retrieval (left panel), fixation durations would be longer on the familiar stimulus relatively to the unfamiliar stimuli, regardless of encoding/retrieval and the number of stimuli. The instructed retrieval hypothesis predicts longer fixation durations in the retrieval conditions only (mid-panel). The lesser exploratory need hypothesis (right panel) predicts longer fixations only when a single familiar stimulus is displayed, but not when it is embedded within other unfamiliar stimuli

As a result, we specifically consider in this paper three hypotheses mentioned above regarding the longer fixation durations: (1) automatic-retrieval/different processing; (2) instructed retrieval and (3) decrease in the need to explore (see Fig. 1). Our novel hypothesis is that longer fixations may be related to a *decrease in the need to explore* a familiar stimulus because a representation of it already exists in memory. In that case, longer fixations would be evident on familiar stimuli only when no alternative stimuli are available to explore. That is, if there is only one image to explore, and the viewer is already familiar with it, fixations should be longer as a result of an attenuated exploratory behavior. However, if there are additional unfamiliar stimuli besides the familiar stimulus, no prolonged fixations are expected—as the observer can explore the other stimuli (see Fig. 1, right panel). If this hypothesis is correct, we expect to find that observers generally tend to look at the familiar stimulus less, and direct their gaze toward unfamiliar stimuli more often and for longer periods of time.

An alternative hypothesis for the longer fixations on familiar stimuli regards *automatic-retrieval/different processing*. It was previously proposed that familiar faces are processed differently due to their memory representations, presumably due to more holistic type of processing (Millen et al., 2020). A related explanation depict that the process of retrieval from memory delays the next saccade and therefore the durations of the fixation become longer (Schwedes et al., 2020). Schwedes et al. argued that while the first fixation is mostly based on information gathering, and planning of the next behavior, the second fixation is lengthened due to the memory retrieval process (Hsiao & Cottrell, 2008; Schwedes & Wentura, 2016; Schwedes et al., 2020). These two hypotheses yield similar predictions in the current study, and therefore joint together. If these two explanations are correct, we expect to find longer fixations regardless of task and the number of stimuli on the display on familiar stimuli (see Fig. 1, left panel).

In addition, we examined what we call the *instructed retrieval* explanation for prolonged fixations. It is possible that lengthened fixations are not due to an automatic process, but rather appear only when participants are required to retrieve information from memory. If that is the case, we expect to find longer fixations when participants are required to retrieve information from memory but not when required to encode information into memory, regardless of the number of images displayed (see Fig. 1, mid-panel).

**Fig. 2:**
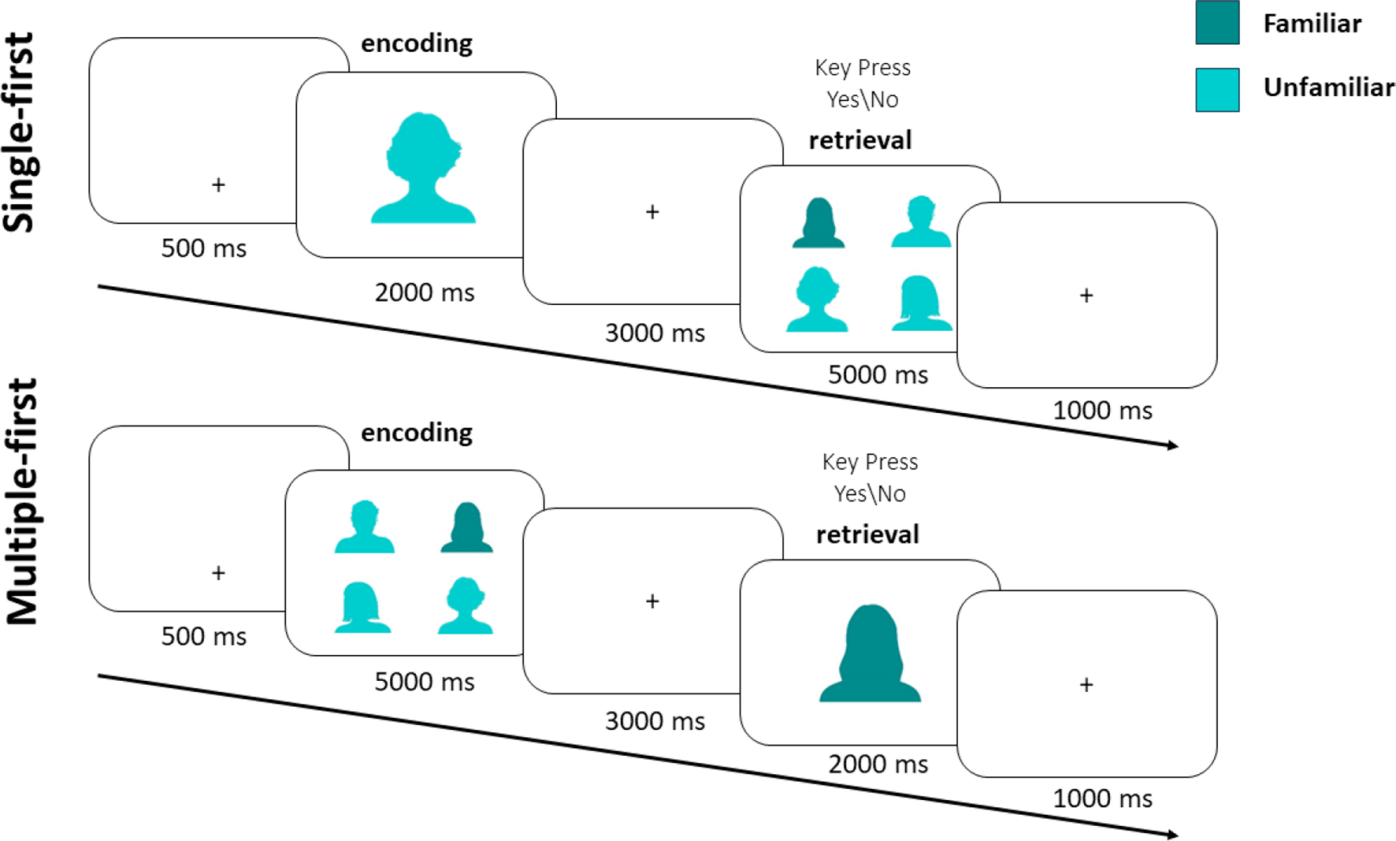
Depiction of the two experimental conditions in the memory task. In the single-first condition, participants were requested to encode a single face, and later report if one of four faces matches the single face they saw before. In the multiple-first condition, participants were requested to encode four faces, and later report if a single face was displayed before as one of the four. For privacy reasons, the silhouettes of the faces are presented instead of the real faces. A familiar face could be presented either during encoding, during retrieval (as in the single-first example) or during the encoding and retrieval (as in the multiple-first example)

In order to test these hypotheses, we designed a study that includes several factors manipulated in a within subject design: one factor relates to the number of stimuli displayed: a familiar stimulus was presented either alone or together with other unfamiliar stimuli. The purpose of these two conditions was to examine the lesser exploratory need hypothesis (see Fig. 2 for an illustration of the task, and Fig. 1 for the three different hypotheses examined). The other, orthogonal, factor relates to the requirement to either encode or retrieve a stimulus from memory—enabling separation between the instructed- and automatic-retrieval hypotheses.

The distinction between these hypotheses will shed light on the mechanism responsible for elongation of fixations on familiar items, arbitrating between distinct mechanisms: whether it is related to different processing and retrieval of information from memory that stalls the fixations, or a need to conserve our valuable muscular saccadic eye movement. Understanding of the factors responsible for prolonged fixations is also significant for the design of Concealed Information Tests (CIT) using eye movements, as it can guide the experimental design and constrain the obtained gaze parameters.

## Methods

All experiments were approved by the ethics committee of the social science faculty in the Hebrew University.

Participants**.** The sample included 59 university students (32 women; Average age 27.7, sd 3.9). Sample size was determined according to the effect size of 0.42 reported in Schwedes & Wentura, 2016. Power of 0.8 lead to sample size of 46 participants following Schwedes & Wentura, 2019 using WebPower package. We kept running participants until the end of the semester in order to reach the desired sample size after exclusions. After exclusion (see criteria below), the sample consisted of 48 participants with normal or corrected-normal vision and valid eye movements data from the entire experiment. All participants signed an informed consent before the experiment. They were granted either course credits or 40 NIS (~ 10$). The experiment was approved by the psychology department ethics committee.

Stimuli**.** We used 64 pictures of past years’ students of the Hebrew University that were held in the University database. All pictures were of neutral expressions, front facing the camera. We normalized the pictures for brightness using a Matlab code (find the code in: https://osf.io/wgfb4/). The stimuli were displayed on a 24″ BenQ 3d monitor, with a 120-Hz refresh rate BenQ monitor and a 1024 × 768 screen resolution, corresponding to a screen size of 47.6° × 28°, situated at a distance of 60 cm from the participants’ eyes.

### Procedure

#### Familiarization stage

The pool of face images was divided randomly into four sets of faces, each composed of eight pictures, four males and four females. At the beginning of the session, each participant memorized one set of images, and sets were counterbalanced across participants. This procedure was used to make sure that familiar faces of one participant would be unfamiliar to other participants. The only difference between familiar and unfamiliar faces was the familiarization stage. First, each image was shown for at least 5 s. Participants could self-pace additional viewing time from the first 5 s onwards if they wanted to spend more time encoding the picture. Next, the 8 familiar faces, along with 8 unfamiliar faces (that were not used later in the experiment) were displayed serially in a random order. To validate that participants remembered the faces, they were asked to indicate by clicking one of two keys if the face is familiar or not. In case they were wrong, the face was repeated later in the session.

#### The memory task

The short-term memory task included two blocks in random order, one with 64 trials of the single-first condition, and the other with 64 trials of the multiple-first condition. Each trial began with a drift check, allowing a deviation of only 0.75 degree of visual angle between the predicted gaze position and the center of fixation point. Larger deviations were accompanied by an error beep and led to a repeated calibration process.

In the multiple-first condition, participants saw a display of four faces (5000 ms), followed by a fixation cross on a blank screen (3000 ms), a single display (2000 ms) and a blank screen with a central fixation cross (1000 ms). During the single face display, participants were required to press one of two keys indicating whether the current face had been presented in the previous multiple display or not.

In the single-first condition, participants saw a single face (2000 ms), followed by a delay with a blank screen and a fixation cross (3000 ms), a multiple display of four faces (5000 ms) and a blank screen with a central fixation point (1000 ms). During the multiple display, participants were required to press one of two keys indicating whether one of the faces had been presented in the previous single display or not. We kept the timings as used in previous published studies (Lancry-Dayan et al., 2018, 2021; Nahari et al., 2019). Parallel displays were displayed longer as they consisted of more faces, leaving more observation time for each face.

Each familiar face appeared in the multiple display in four trials, once in each location of the display (top right, top left, bottom right, bottom left). In the single display, each familiar face appeared twice, once when it had appeared in the multiple display and once when it had not. Since each participant saw 8 familiar faces, and 56 unfamiliar faces, a familiar face appeared in half of the multiple displays (32 trials) and in quarter of the single displays (16 trials). The other multiple displays (32 trials) consisted only of unfamiliar faces, following the same organizing principle of four repetitions for each location of the screen for each face. The faces in both displays (i.e., the multiple and the single ones) were from the same sex, and the correct answer in half of the trials was “yes” and in the other half “no?,” in a random order in both conditions.

At the end of the experiment, participants were rewarded based on their accuracy—if they were correct (accurately reporting if a face in the retrieval display was also displayed in the encoding display) in over 90% of the trials, they were rewarded a bonus (10 NIS, ~ 2.5$).

Participants performed a practice session before each condition block, and had to complete at least three correct practice trials out of five, otherwise, they underwent another session of five training trials.

#### Debriefing questionnaire

After the main memory task, participants completed a debriefing questionnaire in which they viewed images of all the faces displayed in the experiment, numbered from 1 to 64, and were asked to indicate the faces that were included in the familiarization stage prior to the memory task.

#### Exclusion criteria

Based on the final debriefing, and similarly to our previous studies (Lancry-Dayan et al., 2018; Nahari et al., 2019), we removed from the analyses trials that included misclassified faces, based on the following criteria: (1) familiar faces that participants did not report as familiar (2.4%) and (2) unfamiliar faces that participants reported as familiar (18.75%). If more than 16 pictures were removed from the data of a single participant (equivalent to a quarter of the total number of pictures), or more than 1 standard deviation above the mean of the rest of the participants, the data of the participant were excluded from the analysis (11 out of 59 participants). The final sample consisted of 48 participants.

Eye tracking. The experiment began with a standard 9-point calibration and validation procedure provided by Eyelink 1000+ (SR Research Ltd., Mississauga, Ontario, Canada). Average accuracy in the validation procedure ranged between 0.25 and 0.82° of visual angle.

In each trial, four identical rectangle interest areas (size: 360 on 360 pixels; 16.7° on 16.7°) surrounded each one of the four faces in the multiple display, separated horizontally by 55 pixels (2.5°) and vertically by 48 pixels (2.23°). In the single display, an interest area was outlined around the presented face (size: 480 on 480 pixels; 22.3° on 22.3°).

### Data analysis

Fixation parsing. The eye-tracking measures are based on EyeLink’s standard parser configuration: samples were defined as a saccade when the deviation of consecutive samples exceeded 30°/s velocity or 8000°/s^2^ acceleration. Samples gathered from time intervals between saccades were defined as fixations.

Preprocessing. In the fixation analysis, the duration of the first, second and mean fixations directed to each image were extracted for familiar and unfamiliar faces and averaged across all presentation of the face. In the dwell time analysis, all the durations of fixations on familiar and unfamiliar faces were summed, and then averaged first within trial to control for the multiple unfamiliar faces present, and then across all trials of each participant. All data analysis and figures were created using R, dplyr and ggplot2 packages (R Core Team 2021; Wickham 2016; Wickham et al. 2023).

Bayesian analysis. In order to provide additional information regarding the likelihood of the competing hypotheses, we computed Bayes factors (BFs) for the statistical analyses of the fixation and total durations measures by a repeated measures Bayesian ANOVA. In the Bayesian ANOVA, we report the BF_inclusion_, based on all models that include the specific effect (whether one of the main effects or the interaction) compared to all models without these effects (Rouder et al., 2017). We additionally examined the student *t*-tests contrasts with Bayesian t-tests. All the Bayesian analyses were conducted using JASP (JASP team, 2017), using the default priors of the software. It is reported after each of the frequentist statistics as the likelihood of the alternative hypothesis relative to the null, given the data.

## Results

### Task performance

**Fig. 3:**
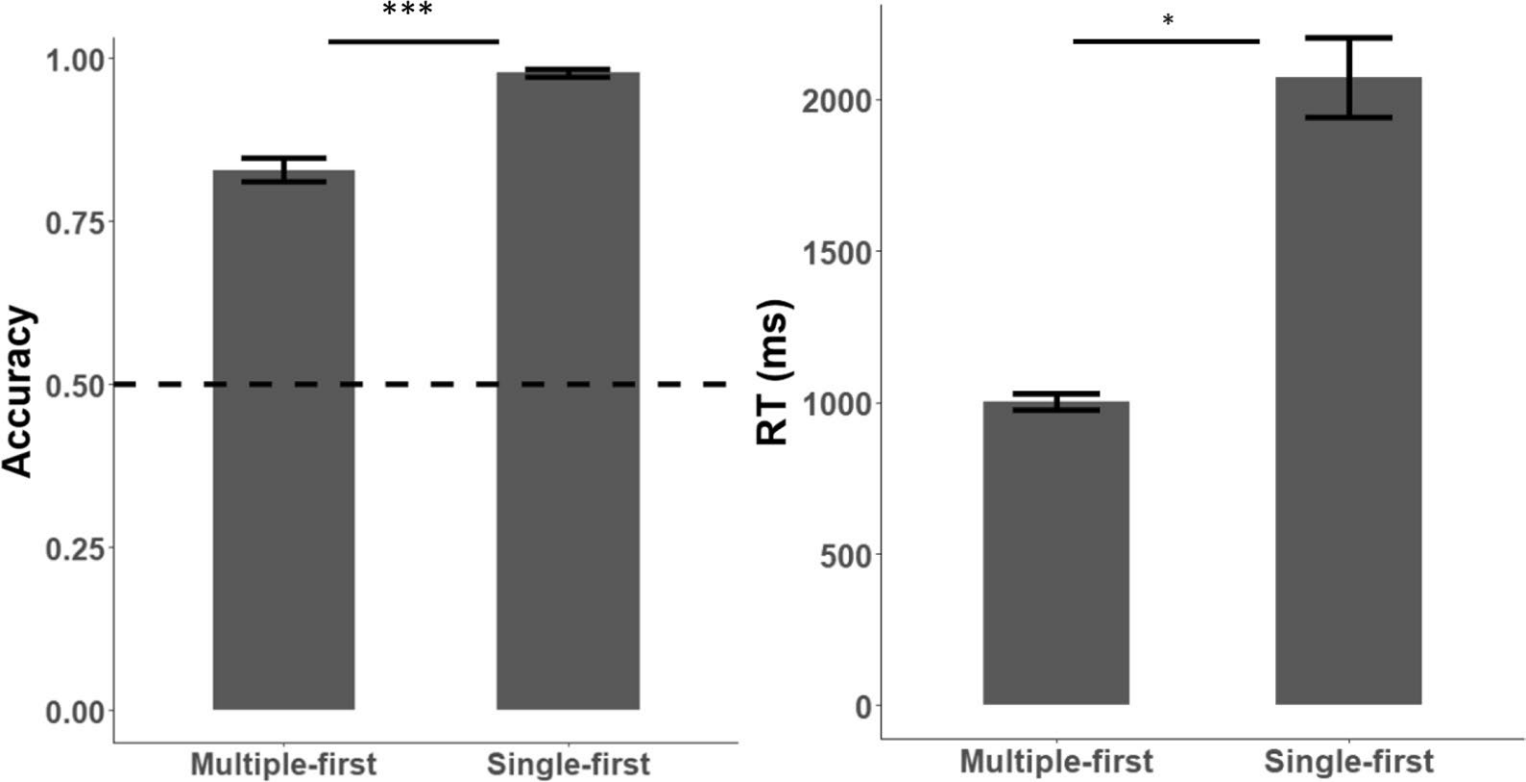
Performance in the short-term memory task. Accuracy rates and reaction times of determining if a face in the first display repeated in the second display. Error bars indicate 1 standard error above and below the mean. *** < 0.001

Participants accuracy in the short-term memory task was better than chance in both conditions (single-first: $t_{48} = 89.28, p < 0.001, d = 12.75$, BF = 14,600 $\times 10^{48}$; multiple-first:$t_{48} = 17.98, p < 0.001, d = 2.57, {\text{BF}} = 6819 \times 10^{14} ;$ Fig. 3). Performance in the single-first condition was better ($t_{48} = 9.03, p < 0.001, d = 1.29,{\text{BF}} = 1404 \times 10^{5} )$), and slower ($t_{48} = 8.81 p < 0.001, d = 1.26, {\text{BF}} = 6872 \times 10^{5} )$) than in the multiple-first condition. In the single-first condition, when the familiar face was the target of the short-term memory task (repeated in the consecutive displays) accuracy levels were higher than when it was an unfamiliar face, without significant differences in reaction times (see supplementary materials for further details; Fig. s1).

### Eye movement analysis

We examined how many fixation's participants performed when observing the faces in the different conditions. The results are summarized in the table (Table 1).

**Table 1:** Summary of the average of number of fixations

Display	Memory task	Average number of fixations	SE
Multiple	Encoding	9.68	0.19
	Retrieval	8.88	0.20
Single	Encoding	4.11	0.12
	Retrieval	3.55	0.11

The duration of the first fixation was examined using repeated measures analysis of variance on the effects of memory task (encoding\retrieval) * familiarity (familiar\unfamiliar) * display (single\multiple). No significant difference was found either between encoding and retrieval (memory task main effect: $F_{1,47}$ = 1.53, *p* = 0.222, $\eta_{p}^{2} = 0.03, {\text{BF}}_{{{\text{inclusion}}}} = 0.023$), nor between single and multiple displays (Display main effect: $F_{1,47}$ = 0.352, *p* = 0.556, $\eta_{p}^{2} = 0.007,{\text{ BF}}_{{{\text{inclusion}}}} = 0.19$), nor between familiar and unfamiliar faces (Familiarity main effect: $F_{1,47}$ = 0.921, *p* = 0.342, $\eta_{p}^{2} = 0.019$, ${\text{BF}}_{{{\text{inclusion}}}}$ = 0.08). Only the interaction between memory task and display was significant, but did not yield higher likelihood than the null model: ($F_{1,47}$ = 5.93, *p* = 0.019, $\eta_{p}^{2} = 0.112$, ${\text{BF}}_{{{\text{inclusion}}}}$ = 0.42). The rest of the interactions, between memory task and familiarity ($F_{1,47}$ = 2.63, *p* = 0.111, $\eta_{p}^{2} = 0.053, \;{\text{BF}}_{{{\text{inclusion}}}} = 0.07$), and the display and familiarity ($F_{1,47}$ = 1.97, *p* = 0.167, $\eta_{p}^{2} = .04, {\text{BF}}_{{{\text{inclusion}}}} = .05$) were not significant. Neither did the three-way interaction between the memory task, display and familiarity ($F_{1,47}$ = 0.07, *p* = 0.794, $\eta_{p}^{2} = 0.001, \;{\text{BF}}_{{{\text{inclusion}}}} = 0.016$) (see Fig. s4 in supplementary materials).

**Fig. 4:**
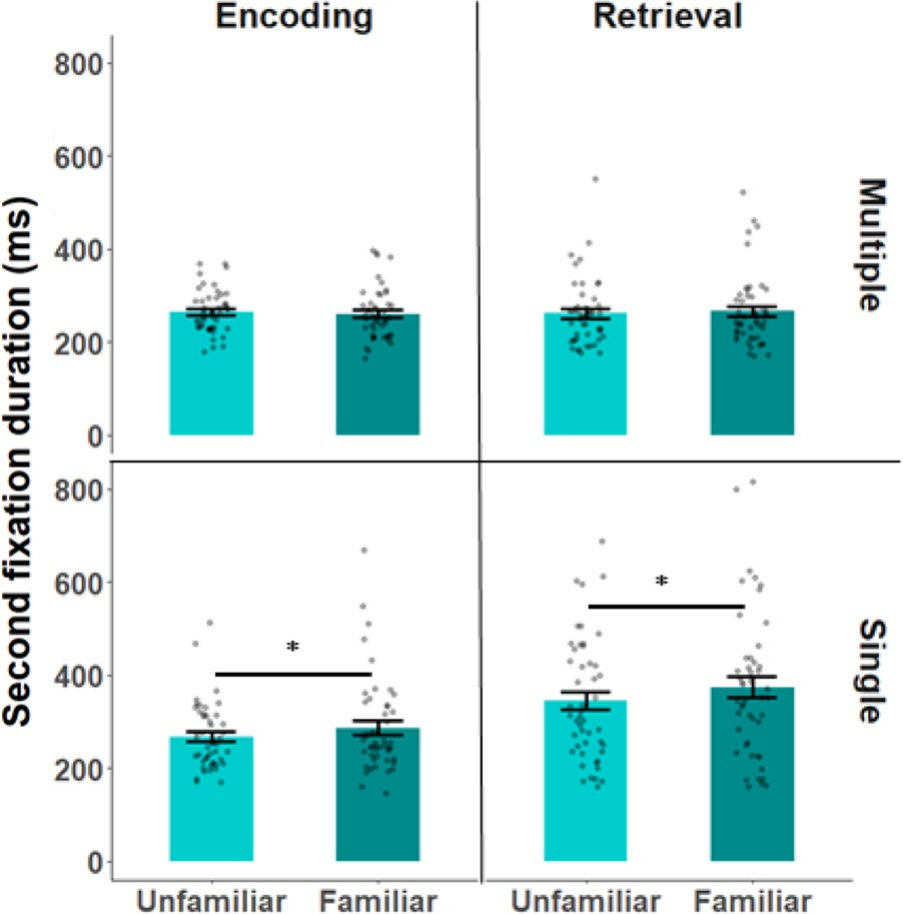
Duration of the second fixation in each of the conditions. Every dot signifies a subject’s mean second fixation duration across all trials of a specific condition: familiar\unfamiliar face, single\multiple display, and encoding\retrieval. Error bars signify 1 standard error above and below the mean. *: < 0.01

Next, we examined the duration of the second fixations (see Fig. 4). All three main effects were significant: the memory task ($F_{1,47} = 16.67, p < 0.001, \;\eta_{p}^{2} = 0.262, {\text{BF}}_{{{\text{inclusion}}}} = 13,961),$), display ($F_{1,47} = 26.22, p < 0 .001, \eta_{p}^{2} = 0.358. {\text{BF}}_{{{\text{inclusion}}}} = 91,6419$) and familiarity $F_{1,47} = 5.95, \;p = 0.019$, $\eta_{p}^{2} = 0.112, {\text{BF}}_{{{\text{inclusion}}}} = 3.518$. The interaction between the display type and memory task ($F_{1,47} = 17.834, p < 0.001$, $\eta_{p}^{2} = 0.275, {\text{BF}}_{{{\text{inclusion}}}} = 501.67$), and between the display and familiarity were significant ($F_{1,47} = 10.44, p = 0.002, \eta_{p}^{2} = 0.182, {\text{BF}}_{{{\text{inclusion}}}} = 5.94)$, but the interaction between memory and familiarity was not ($F_{1,47} = 0.726, p = 0.399, \eta_{p}^{2} = 0.015, {\text{BF}}_{{{\text{inclusion}}}} = 0.74$). The three-way interaction between the memory task, display and familiarity was not significant ($F_{1,47} < 0.001, p = 0.978$, $\eta_{p}^{2} = < 0.001, {\text{BF}}_{{{\text{inclusion}}}} = 0.784$).

Planned contrasts to compare the effect of familiarity when multiple faces were displayed were not significant for either encoding ($\psi = 4.16, \;t_{175} = 0.44,\;p = 0.658, {\text{BF}} = 0.086$) or retrieval ($\psi = 4.81, \;t_{175} = 0.51,\;p = 0.609, \;{\text{BF}} = 0.268$). On the other hand, when a single face was displayed, significantly longer fixation durations were observed on the familiar face (encoding: $\psi = 19.14,\; t_{175} = 2.04,\;p = 0.043, \;{\text{BF}} = 3.44$ and retrieval: $\psi = 28.63, \;t_{175} = 3.05,\;p = 0.003,\;{\text{ BF}} = 1.97;$ see Fig. 4).

Next, we examined the mean fixation duration across all fixations on the stimulus (Fig. s5 in the supplementary materials). The main effects of memory task (encoding\retrieval): ($F_{1,47}$ = 8.11, *p* = 0.007, $\eta_{p}^{2} = 0.157, \;{\text{BF}}_{{{\text{inclusion}}}} = 60.1$) and display (multiple\multiple: $F_{1,47}$ = 24.57, *p* =  < 0.001, $\eta_{p}^{2} = 0.343,\;{\text{ BF}}_{{{\text{inclusion}}}} = 15683.49$) were significant, but not familiarity (familiar\unfamiliar: $F_{1,47}$ = 3.725, *p* = 0.06, $\eta_{p}^{2} = 0.07,\;{\text{ BF}}_{{{\text{inclusion}}}} = 0.37$). The interaction between memory and display was significant: ($F_{1,47}$ = 10.93, *p* = 0.002, $\eta_{p}^{2} = 0.19,\; {\text{BF}}_{{{\text{inclusion}}}} = 38.3$), but the rest of the interactions were not (memory task × familiarity $F_{1,47}$ = 1.83, *p* = 0.201, $\eta_{p}^{2} = 0.03,\;{\text{ BF}}_{{{\text{inclusion}}}} = 0.36$; display × familiarity $F_{1,47}$ = 0.22, *p* = 0.64, $\eta_{p}^{2} = 0.005,\;{\text{ BF}}_{{{\text{inclusion}}}} = 0.24$). Neither did the three-way interaction between the memory task, display and familiarity ($F_{1,47}$ = 1.02, *p* = 0.318, $\eta_{p}^{2} = 0.02,\;{\text{ BF}}_{{{\text{inclusion}}}} = 0.17$).

### Dwell time analysis

The fixation duration analysis shows that the second fixations were elongated when a single familiar face was displayed, relatively to unfamiliar faces, supporting the less exploratory need hypothesis. If the effect of fixation duration disappears in the multiple display because there are other, unfamiliar, faces to explore, this should be evident also in the overall dwell time gaze is directed to the faces. We expected that participants would spend less time looking at the familiar face during the encoding phase of the short-term memory task. This expectation is based on the idea that participants would prioritize encoding the unfamiliar faces, as these new faces require more cognitive resources to be successfully reported later (Jackson & Raymond, 2008). Since the familiar face is already known, there is less need to focus on it, leading to shorter dwell times (Nahari et al., 2019). Furthermore, we anticipated observing a similar, though less pronounced, effect during the retrieval phase. Even at this stage, participants are likely to spend more time exploring the unfamiliar faces in the display, as these faces take more time to encode and thus require additional processing to accurately complete the task (Ramon & Gobbini, 2018). On the other hand, the familiar faces receive enhanced processing efficiency, allowing participants to allocate more time to the unfamiliar faces to improve task performance (Nahari et al., 2019; Ryan et al., 2007).

**Fig. 5:**
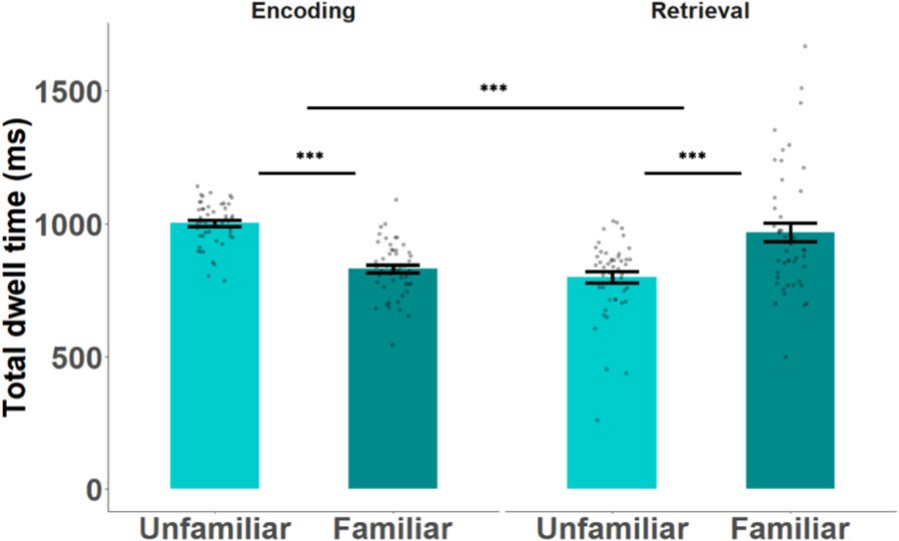
Total dwell time on the different faces during the multiple four face display. The unfamiliar bars show averages across the three unfamiliar faces. Error bars indicate 1 standard error above and below the mean. ***: < 0.001

The main effects of familiarity ($F_{1,47} = 0.004, \;p = 0.948, \eta_{p}^{2} < .001,\;{\text{ BF}}_{{{\text{inclusion}}}} = < 0.001)$ and memory task ($F_{1,47} = 2.242, \;p = 0.141,\; \eta_{p}^{2} = 0.04,\;{\text{ BF}}_{{{\text{inclusion}}}} = < 0.001)$) were insignificant; however, the interaction between the two was significant ($F_{1,47} = 89.576, \;p < 0.001, \;\eta_{p}^{2} = 0.65,\;{\text{ BF}}_{{{\text{inclusion}}}} = 1407 \times 10^{10} ;$ see Fig. 5). Direct contrasts revealed significant differences between the total dwell time on familiar and unfamiliar faces within each memory task: (encoding: $\psi = 170.9,\; t_{90} = 6.$ 02, $p < 0.001$, BF = $2341 \times 10^{4}$, retrieval: $\psi = 189.9, \;t_{90} = 5.92,\;p < 0.001, \;{\text{BF }} = { }1348.9$). This was evident also when excluding the time following the key press (see supplementary material Fig. s3).

A similar pattern was reflected also in the number of fixations. Fewer fixations during encoding of a familiar face were observed, in comparison to unfamiliar faces, as shown by the three-way interaction between the memory task, display and familiarity was significant ($F_{1,47} = 44.04,\; p < 0.001,\; \eta_{p}^{2} = 0.484, \;{\text{BF}}_{{{\text{inclusion}}}} = 5521 \times 10^{6}$). For further details on the number of fixations analysis, see supplementary materials (Fig. s2).

## Discussion

Significant differences were observed between the two conditions, parallel-first and single-first in terms of both reaction time and memory accuracy. These are related to the efficiency of encoding faces; evidently, it is easier to compare the internal representations of four faces to one single image presented visually, than visually examine four faces and compare them to one stored in memory.

Consistent with previous studies, when participants looked on familiar faces presented alone, their second fixations were longer (Hsiao & Cottrell, 2008), but not when other unfamiliar faces were displayed simultaneously. It may indeed be the case that during retrieval, the second fixation duration on single displays reflect a recollection processes, and\or holistic perception, as suggested previously (Millen et al., 2020; Schwedes et al., 2020). However, the similar fixation durations during the multiple displays, in which unfamiliar faces were displayed together with the familiar face, suggests that the differences in durations are dependent not only on familiarity, but also on the need and motivation to explore, shaped by the availability of other stimuli in the display. We speculate that previous studies have found lengthened fixations since the stimuli were singly presented, or, that the task explicitly required retrieval from memory. In the current study, the task was unrelated to the long-term familiarity, and consequently, no elongation was observed when multiple faces were displayed.

Another consistent difference in gaze pattern was found in the multiple-face displays. There, overall dwell time was shorter on the familiar face compared to unfamiliar faces during encoding, but longer during retrieval. The shorter overall dwell time on familiar faces is consistent with our lesser need to explore hypothesis and reflects the need to explore unfamiliar stimuli. This effect is also consistent with previous studies (Lancry-Dayan et al., 2018) and is adaptive as it is easier to encode familiar stimuli into short-term memory, encouraging deployment of resources to unfamiliar faces (Nahari et al., 2019). Unlike our prediction, during retrieval, gaze was directed more toward familiar faces than unfamiliar faces. We speculate that this effect is due to a confusion between long-term and short-term memory, in which subjects may not be sure what is the encoding source of the current faceṣ—the previous encoding stage from five seconds ago, or the initial familiarity stage that occurred at the beginning of the experiment. We hypothesize that such confusion may hamper the short-term memory reports and lead to longer dwell time on the face, and could be tested. Future studies could employ a source monitoring paradigm (Johnson et al., 1993), where faces are presented at varying intervals, and participants are periodically asked to recall or recognize faces from specific stages of the task (i.e., identify whether a face was seen during the initial familiarity stage or during a recent encoding trial). This would allow researchers to examine how well participants can distinguish between memories formed at different times and how this ability influences their gaze behavior and task performance.

From an applied perspective, our results support the potential contribution of fixation duration to memory detection, provided that the familiar and unfamiliar stimuli are displayed serially. However, it also signifies the importance of using multiple measurements when testing for concealed information, to increase the validity of the measure (Klein Selle et al., 2016; Millen et al., 2020). Many measurements have been proposed before, as well as combinations between them, in order to create a better detection score (Meijer et al., 2014). Notably, when trying to detect concealed information, it is better to use multiple measures (Lancry-Dayan et al., 2021; Meijer et al., 2014; Millen et al., 2020). Our study shows that the second fixation duration could be used as another marker of recognition depending on the task and display.

A limitation in the current study is the relatively large number of misclassified pictures. At the end of the experiment, participants were asked to indicate which faces they had learned at the beginning of the experiment. This final memory test had a relatively large error rate (21.15%), with unfamiliar faces either misclassified as previously learned or learned faces classified as unfamiliar (see Fig. s6 in the supplementary materials). We believe that this confusion is due to the relatively long task, which lasted about an hour and included 128 trials of short-term memory queries. Future research could tackle this issue by employing a longer familiarization phase, a deeper encoding method (Schwedes et al., 2020; Sporer, 1991), or a between-subjects design that will allow showing only one condition, either multiple-first or single-first, thus shortening in half the experiment duration. In any case, the effects reported here are robust and can be seen even when the analyses include all faces without exclusion (see Figs. s7–s9). Another thing to consider is that the faces in the multiple displays were smaller than the single faces, which may mask the effects of holistic processing. Future studies should examine the effect of stimuli size on fixation durations on familiar stimuli. In addition, it is worth noting the difference in sizes between the multiple and the single display. Since the single-display face is larger, parafoveal processing is limited compared to the parallel. This may affect the results by inducing more fixations with decreased duration. However, most of the second fixations fall within a portion of the single face that is proportional to the size of the multiple display face, and therefore we believe the comparison is valid (see supplementary Fig. s10).

There are numerous future research directions that emerge from the theoretical hypothesis supported by the current results. An interesting question regards the limits to the need of exploration, for example, by manipulating the number of total stimuli on display (by extending the range between 1 and 6, as been previously used), and the number of familiar pictures amongst them. This would allow further clarification of when exactly reduced exploration is exhibited. In addition, examining individual differences within this effect would elaborate on how different individuals engage in visual exploration differently. If individuals differ in their exploratory gaze behavior in a consistent manner, it would be interesting to examine the association of this behavior with other related personality traits, such as curiosity. Curiosity is expected to generate stronger exploratory urges regardless of familiarity, and is therefore expected to correlate negatively with the effect. It would also be of importance to understand whether the strength of the memory representation is related to the effect magnitude, for both theoretical and applicative reasons (i.e., in cases of detection of information). Another way to examine the lesser need of exploration would be to generalize it in a more ecological setup, such as by using virtual reality. Virtual reality setups enable participants to change their head position, see full body figures and even different scenes. It is not clear if such experimental differences would influence the time that participants engage in looking at familiar and unfamiliar faces. Lastly, comparing directly between tasks that require explicit recognition a tasks that do not demand memory at all, like a target detection (Nahari et al., 2019) is warranted, as all current tasks were based on memory, thus implicitly demanding the involvement of it on the deployment of visual exploration.

Another avenue of research regards the spatial distribution of fixations, which are more prevalent in domains like scene perception (Greene & Oliva, 2009) and saliency algorithms (Itti & Koch, 2001). Previous studies had examined fixation distributions in the context of memory tasks, and found similar effects—the longer the participants had viewed the scene, the fixation rate decreased but the fixation duration increased (Lancry-Dayan et al., 2019). In addition, previous findings suggested differences in the fixation patterns of personally familiar faces. For example, a more distributed pattern of fixations on unfamiliar faces (Ramon & Gobbini, 2018), or a differentiation that occurs from the second fixation onwards, showing a tendency to focus on more local features in the familiar faces compared to the unfamiliar (Van Belle, 2010). However, we did not fully replicate these results (see Fig. s11). Regarding the multiple-face display, it will be of interest to examine how manipulations of the number of familiar faces within the display alters the spatial distribution of the fixations, as it makes sense that these will affect the scanning patterns and overall distribution of fixations. We hypothesize that displays with a higher number of familiar faces will result in less spatial exploration by participants due to the enhanced cognitive processing allocated to each familiar face (Ramon & Gobbini, 2018). This relationship is contingent on the task at hand and may change if the task requires ongoing exploration, such as in a dot detection task as opposed to a memory task (Nahari et al., 2019)).

To conclude, the finding that the second fixation on a familiar stimulus is longer when it is displayed alone, points to a mechanism that takes into account not only the current visual stimulus and its presence in memory, but also the competition from other stimuli around it. These findings highlight the role of memory in shaping active sensing and bring forward constraints, and promise, in using fixation durations as markers for recognition in concealed memory tests.

## Supplementary Information

Supplementary Material 1

## Data Availability

The datasets generated and/or analyzed during the current study will be available upon request once the paper is published.
