# Supplementary material for: Fixation durations on familiar items are longer due to attenuation of exploration
Source: Cogn Res Princ Implic. 2024 Nov 14;9:77. doi: 10.1186/s41235-024-00602-5 (PMC11564497; doi:10.1186/s41235-024-00602-5)
Supplement: Supplementary file 1 — Supplementary Material 1 [file 41235_2024_602_MOESM1_ESM.docx]

**Supplementary materials**

**Behavioral measures in the two experimental conditions by familiarity.**

In the main text we report the accuracy and reaction time of the short-term memory task in the two conditions separately – the multiple first and the single first. Here we completement this analysis by taking into account the familiarity with the stimuli. Here, familiarity is defined according to whether a familiar stimulus was present during the time that demanded the choice (key press). In other words in the multiple-first condition, familiarity is defined by whether the single face was familiar to the subjects. In the single-first, familiarity was based on whether a familiar face appeared in the multiple four face display.

Figure s1. Right panel depicts accuracy across the multiple-first and single-first conditions, depending on familiarity. On the left, RT during the two conditions. Darker color indicated the familiar face.

We performed an rANOVA to see how the reaction time is influences by the task (multiple\single-first) and familiarity (familiar\non familiar). The only significant effect was the main effect of the task ($F_{1,47}=77.97, p<.001, \eta_{p}^{2}=.62, BF_{inclusion}=1429\times{10}^{4})$. neither the effect of familiarity ($F_{1,47}=0.002, p=0.965, \eta_{p}^{2}<.001, {BF}_{inclusion}=.14)$ or the interaction between familiarity and task ($F_{1,47}=0.05, p=.823$, $\eta_{p}^{2}<.001$, ${BF}_{inclusion}=.13$) were significant.

We additionally examined how accuracy is modified by the task (multiple\single-first) and familiarity (familiar\non familiar), by an additional rANOVA. Here, all three effects were significant: task ($F_{1,47}=65.53, p<.001, \eta_{p}^{2}=.58, BF_{inclusion}=2368\times{10}^{4})$, familiarity ($F_{1,47}=6.06, p=0.018, \eta_{p}^{2}=.11, BF_{inclusion}=8.2)$ and the interaction between familiarity and task ($F_{1,47}=7.86, p=.012, \eta_{p}^{2}=.12, BF_{inclusion}=18.3$). It is important to note in this context the ceiling effect in the “single-first” condition, which might mask the familiarity effects in this condition. Overall, it is evident that when the familiar face was present, it benefited the correct responses in the multiple-first condition, without hampering the reaction time.

Finally, we examined the length of the experiment as an influencing factor on task performance. In order to examine time-based effects, we ran a logistic-mixed model analysis, regressing accuracy on task, block and trial index (accuracy ~ block + trialIndex + task + (1 | subject)). Indeed, there was a negative effect of block (b = -0.3, z = -2.44, p = 0.01), indicating that as blocks progressed, the accuracy dropped, showing the hypothesized effect of time over the accuracy, and a positive effect of the task (Where single first had more accurate responses: b=2.28, z = 12.89, p<.001), there was no effect of the trial index (b=-0.002, z = -0.41, p = 0.678). the intercept was significant (a = 2.37, z = 8.79, p<.001), indicating that overall participants were more accurate than inaccurate responses.

To conclude, there was an effect of the length of the study on short-term memory performance, which might have also impacted long-term memory performance. Therefore, it is important to keep the experiment and concealed information tests duration shorter, especially during applied settings.

**Number of fixations analysis in the multiple and single displays.**

The main text focuses on fixation durations, here we report a complementary measure - the number of fixations and examine whether it is altered by familiarity and display type. For that purpose, we performed a repeated measure three-way ANOVA of memory task (Encoding\retrieval), familiarity (familiar\unfamiliar), and display type (Multiple\single). All three main effects were significant: the memory task ($F_{1,47}=57.26, p< .001, \eta_{p}^{2}=.55, {BF}_{inclusion}=6565\times{10}^{9}),$display ($F_{1,47}=446.52, p< .001, \eta_{p}^{2}=.9,{BF}_{inclusion}=6565\times{10}^{9}$), and familiarity ${(F}_{1,47}=27.54, p< .001, \eta_{p}^{2}=.37,BF_{inclusion}=6565\times{10}^{9})$. The interactions between the display type and memory task ($F_{1,47}=32.34, p< .001, \eta_{p}^{2}=.41, BF_{inclusion}=9249\times{10}^{9}$), and between the task and familiarity were significant ($F_{1,47}=51.13, p< .001, \eta_{p}^{2}=.52, BF_{inclusion}=7282\times{10}^{9})$, but the interaction between display and familiarity was not ($F_{1,47}=.525, p= .472, BF_{inclusion}=8032\times{10}^{5}$). The three-way interaction between the memory task, display and familiarity was significant ($F_{1,47}=44.04, p< .001, \eta_{p}^{2}=.48, BF_{inclusion}=5521\times{10}^{6}$). These results indicate less fixations on the familiar face in all conditions but the retrieval-multiple. This shows that there is a lesser need of sampling the familiar face, which mimics the result with fixation duration, interestingly found here in the multiple-encoding condition too. The opposite result of more fixations during retrieval could indicate a source confusion between long- and short-term memory when identifying the face.


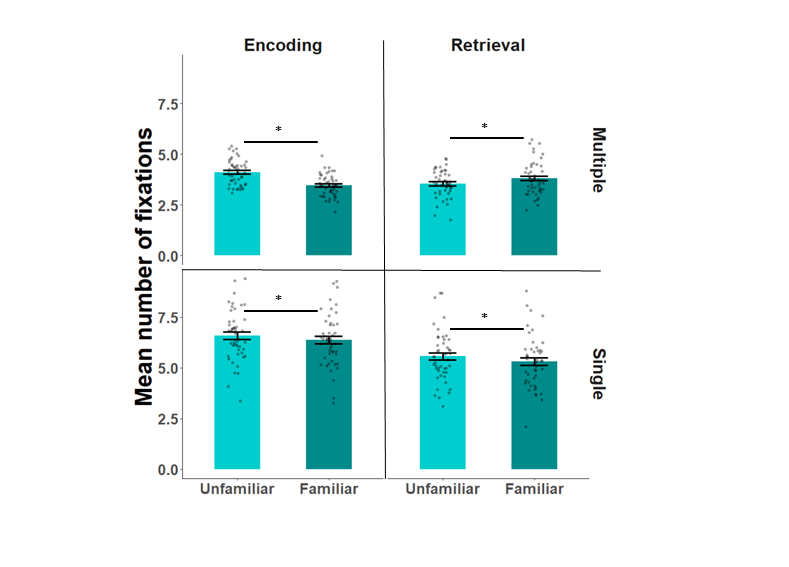


Figure s2. Mean number of fixations across the different conditions, all yielded significant effects.

**Analysis of the total dwell time before the key press.**

Participants required to indicate by a key press during the retrieval phase of the multiple display whether the face appeared in the previously encoded face. In order to make sure that the extra time after completing the task did not alter the results since the task after a decision is reached is different, and might not be similar to the task before the press, we analyzed the total dwell time based on the familiarity only until the key was pressed.

This analysis reveals the same interaction as indicated by using the complete trial time, and also shows significant effects of the task and familiarity. However, since the trial time was shortened due to the button press during retrieval, but not during encoding that demands all the time, it is obviously expected to see a main effect of task ($F_{1,47}=240.66, p<.001, \eta_{p}^{2}=.692, BF_{inclusion}=1737\times{10}^{13}$). Despite the lack of effect of familiarity ($F_{1,47}=0.51, p=0.478, \eta_{p}^{2}<.001, BF_{inclusion}=0.2)$, there was a strong interaction effect between the two ($F_{1,47}=196.30, p<.001, \eta_{p}^{2}=.10, BF_{inclusion}=1998\times{10}^{16}), similarly to the results of the entire trial time .$Contrasts of the memory process showed a significant effect for both the encoding ($t_{47}=7.74, p<.001, BF=2341\times{10}^{4})$ and retrieval ($t_{47}=8.89, p<.001, BF_{10}=7907\times{10}^{3}$). To conclude, the analysis of total dwell time while considering only the time before the key press replicated the same findings, showing a significant interaction effect between familiarity and the task.


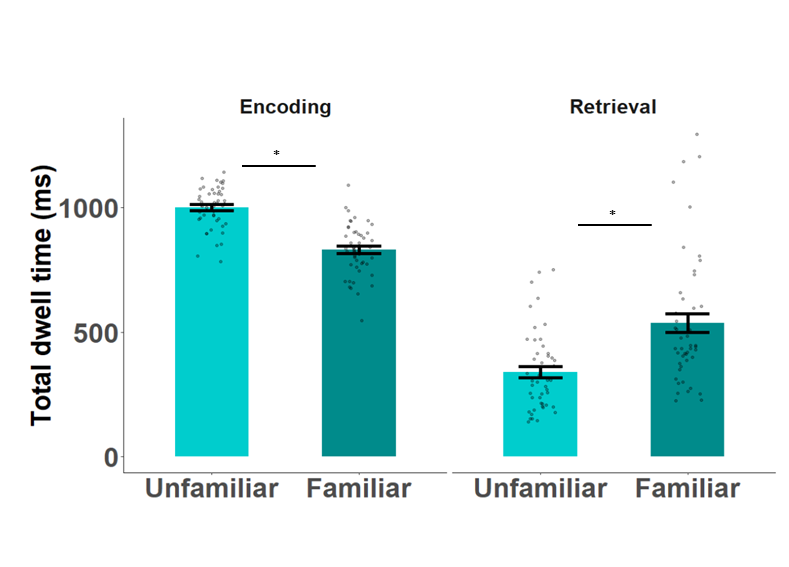


Figure s3. Analysis of the total dwell time, including the memory task encoding and retrieval and familiarity. *: p<.05.

**Analysis of the first fixation duration.**

Previous research has found inconsistencies in the first fixation duration on familiar faces – some found an effect (Ryan et al., 2007b), but other did not (Schwedes & Wentura, 2012). The first fixation duration effects were analyzed, but revealed no statistical differences in either of the main effects or the interactions with familiarity (see main text). As other researchers have suggested, the first fixation might be more related to low-level perceptual features (Schwedes & Wentura, 2016b), and therefore does not drive the memory related effects.


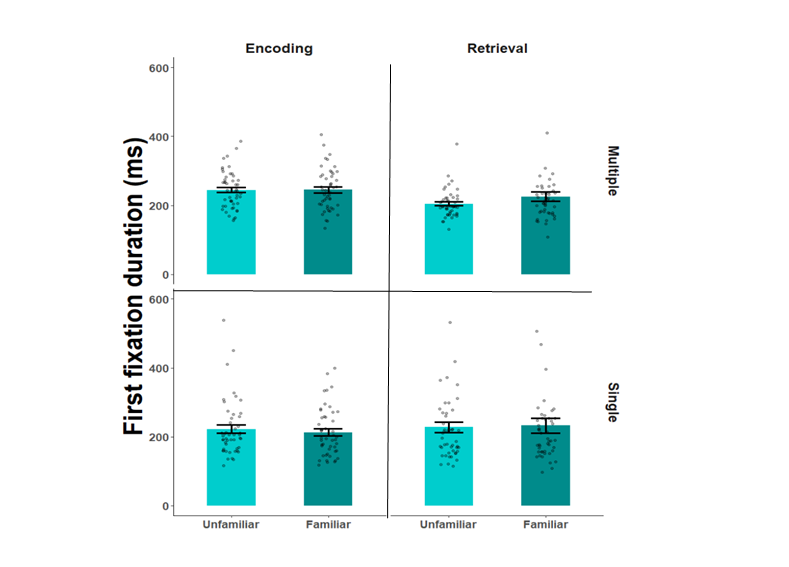


Figure s4. First fixation durations across the difference experimental conditions: display, task and familiarity.

**Analysis of the mean fixation duration.**

Previous research has found the mean fixation duration to be longer on familiar items (Lancry-Dayan et al., 2018a; Millen & Hancock, 2019; Peth et al., 2013). Therefore, we examined the mean fixation duration as well, which yielded no significant interactions with familiarity (see main text for full results). However, no significant effects of interest were found, which could indicate the instability of the measure. It could be that the effect of the average fixation duration was only due to the second fixations, and therefore is less stable.


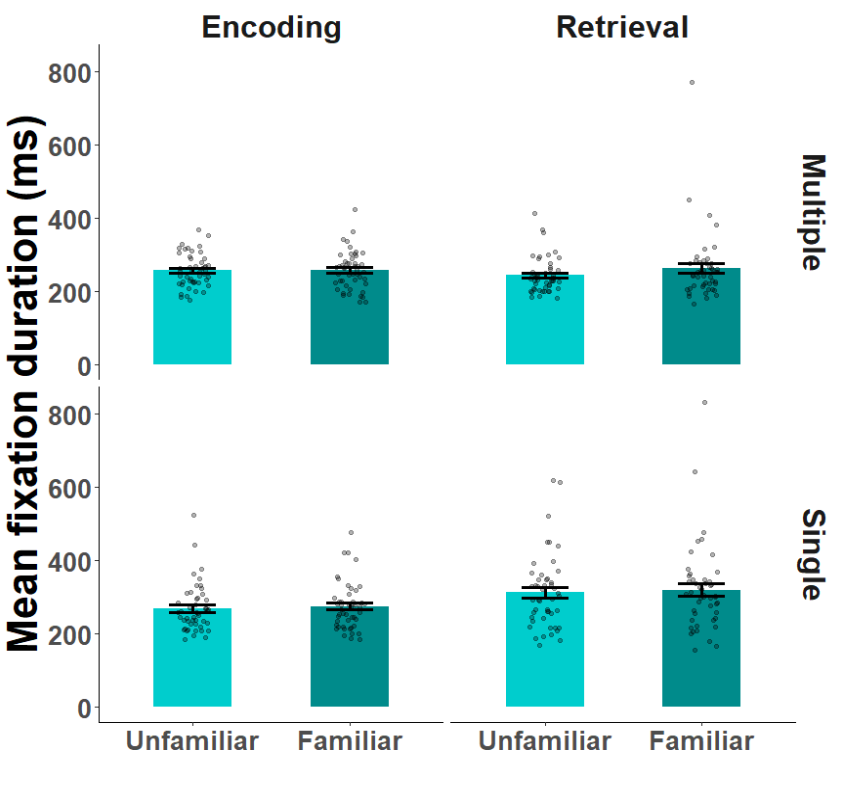


Figure s5. Mean fixation duration across the difference experimental conditions: display, task and familiarity.

**Results without participants’ and pictures removal**

There were many misclassified pictures in the dataset (see methods), that led to disqualifying some of the participants (see figure 5). In order to check the robustness of the effect, we repeated the main analysis from the paper without participants’ exclusion.


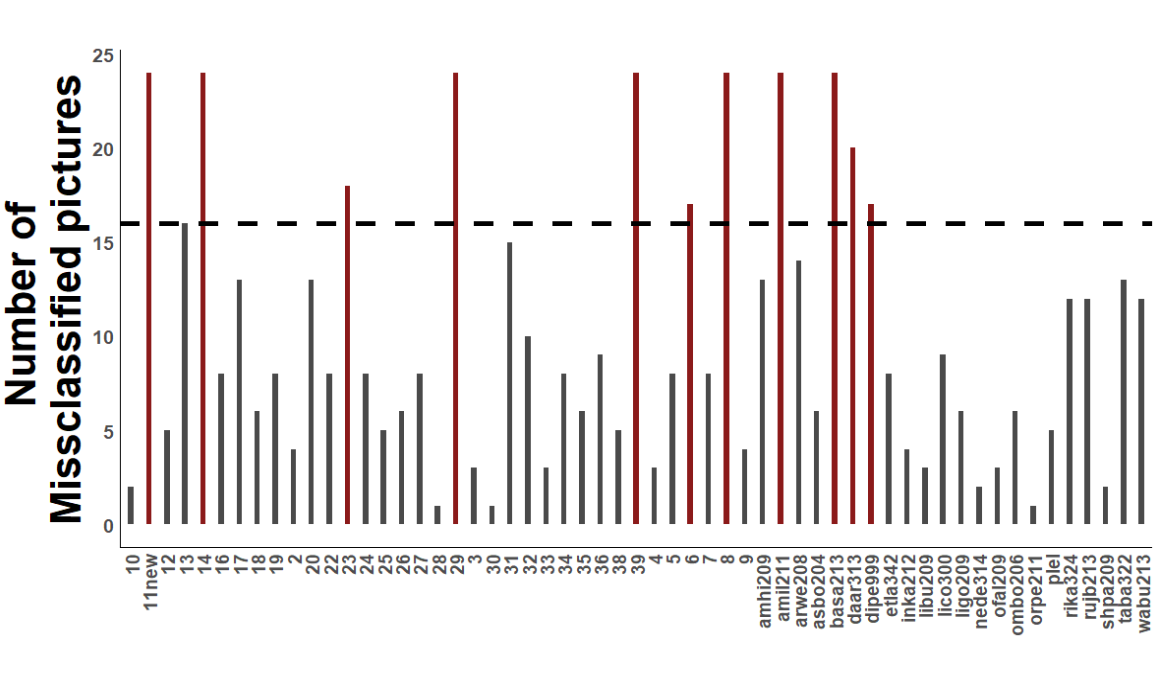


Figure s6. Number of misclassified pictures by subject (either forgotten or wrongly marked as familiar). Each bar represents a subject. The dashed line marks 16 pictures, the cutoff. Subjects’ colored red were removed from further analysis.

We performed an rANOVA without exclusion of participants or pictures, seeing whether the second fixation duration is influenced by the effects of memory processing condition (encoding\retrieval) * familiarity (familiar\unfamiliar) * display (multiple and single). All three main effects were significant: the memory task ($F_{1,58}=14.25, p< .001, \eta_{p}^{2}=0.19,BF_{inclusion}=12529),$display ($F_{1,58}=30.31, p< .001, \eta_{p}^{2}=0.34, BF_{inclusion}=4215\times{10}^{2}$), and familiarity ($F_{1,58}=7.31, p= .009, \eta_{p}^{2}=0.11, BF_{inclusion}=1.74)$. among which the familiarity effect had only minor support in favor of the effect. The interactions between the display type and memory task ($F_{1,58}=18.88, p< .001$, $\eta_{p}^{2}=.246$, $BF_{inclusion}=773.9$), and between the display and familiarity were significant ($F_{1,58}=5.31, p= .025, \eta_{p}^{2}=.084, BF_{inclusion}=1.7)$, but the interaction between memory and familiarity was not ($F_{1,58}=.07, p= .782, \eta_{partial}^{2}=.001, BF_{inclusion}=.352$). The three-way interaction between the memory task, display and familiarity was not significant ($F_{1,58}=.79, p= .375, \eta_{p}^{2}=.01$, .352).

Planned contrasts to compare the effect of familiarity within each condition were performed. While the contrasts were not significant for either of the multiple displays (Encoding: $\psi=0.16, t_{229}=0.02,p=.984$, $BF_{10}=.14$*,* retrieval: $\psi=5.17, t_{229}=0.64,p=.52, BF_{10}=.17$). However, within the single displays, the results were significant for encoding, showing longer fixation durations on the familiar faces (Encoding: $\psi=24.5, t_{229}=3.04,p=.003, BF_{10}=14.07)$ and marginally significant, inconclusive results for the retrieval: ($\psi=14.67, t_{229}=1.82,p=.07, BF_{10}= .31$).


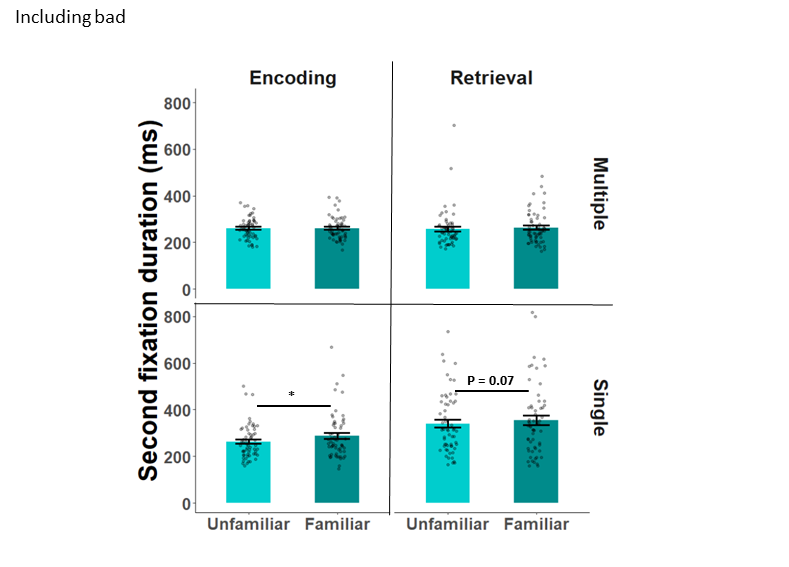


Figure s7. Results of the second fixation duration without subjects’ or pictures removal.

Next, we examined the total dwell time results across the entire cohort of participants and pictures, the rANOVA of memory task and familiarity yielded a main effect of the task ($F_{1, 59}=5.39, p=0.024, \eta_{p}^{2}=.085, BF_{inclusion}=.793)$. without an effect of familiarity ($F_{1, 59}<.001, p=1.0,\eta_{p}^{2}<.001, BF_{inclusion}-.163)$, but a strong interaction effect between the two ($F_{1, 59}=145.39, p<.001, \eta_{p}^{2}=.715, BF_{inclusion}=1649\times{10}^{18})$.


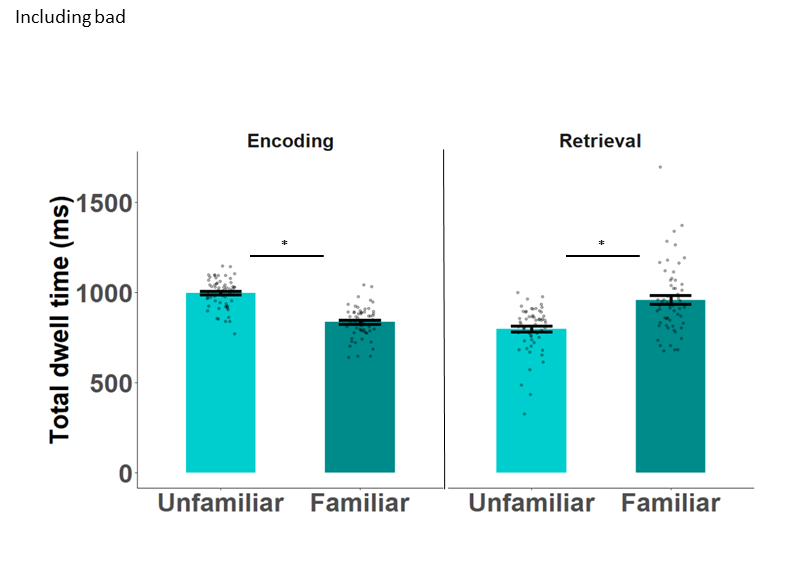


Figure s9. Results of the total dwell time analysis without exclusion of participants’ or pictures.

To conclude, the results of the analysis without removing subjects of pictures who were misclassified replicated most of the effects, which contributes to the robustness of the results.


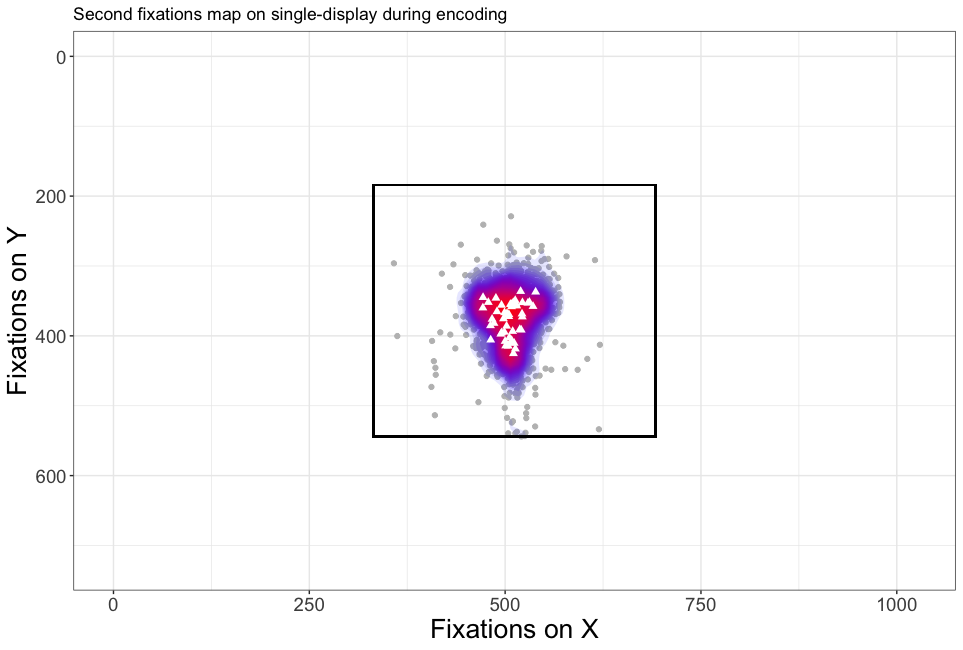

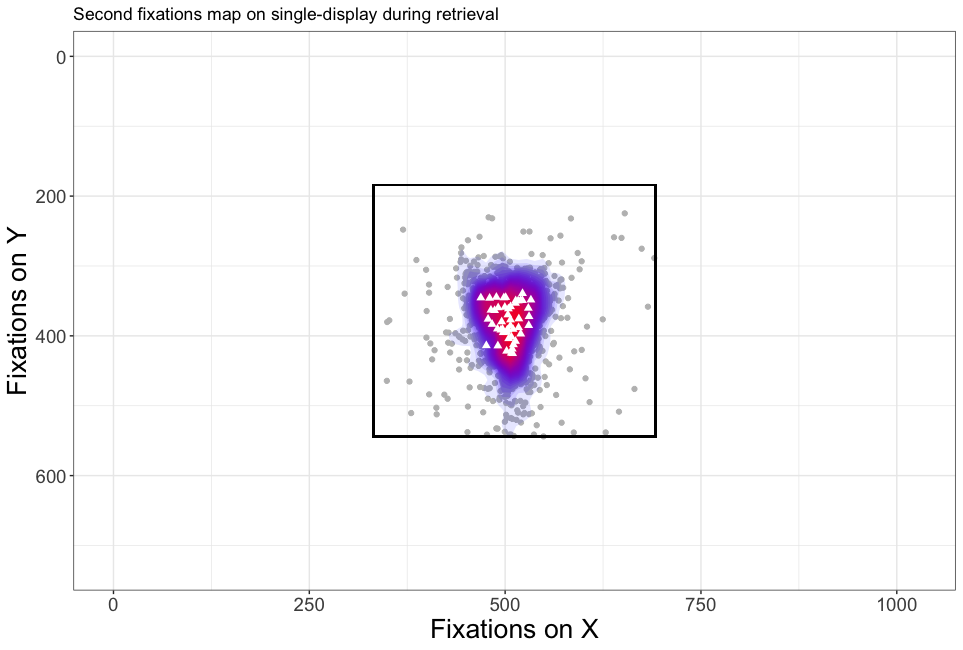


Figure s10. Fixation maps of the second fixation on the single-face display, face bounderies are in black frame. Average location of second fixation duration per participant are shown as white triangles. Both encoding and retrieval show that the second fixation locations fall within a range of approximately 300 px (13.9° visual angle) on the Y-axis and 200 px (9.2° visual angle) on the X-axis. Top: encoding. Bottom: Retrieval.

The figure demonstrates that a majority of second fixations fall within the boundaries of the size of the multiple face display as well (equivalent to 360 on 360 pixels; $16.7^{\circ}$ on $16.7^{\circ}$), therefore the comparison we make between the fixations of the single and the multiple displays are valid in terms of parafoveal processing.

**Analysis of root mean squared distance.**

We tested the fixation location using t-tests to compare between the average differences in the root mean square distance (RMSD) from the center of the screen (512, 384) between familiar and unfamiliar trials.

In the single display we found a significant effect during encoding: (t_47_=3.01, p=0.004); but not during retrieval: (t_47_ =0.96, p = .34), in both cases the distances were larger when observing the unfamiliar faces rather than a familiar one. However, in the multiple displays, we found a significant effect during retrieval (t_47_ = 2.98, p = .004), but with longer distance in the familiar multiple displays than the unfamiliar ones. We did not find an effect during the encoding of the multiple displays: t_47_=0.66, p=0.51).


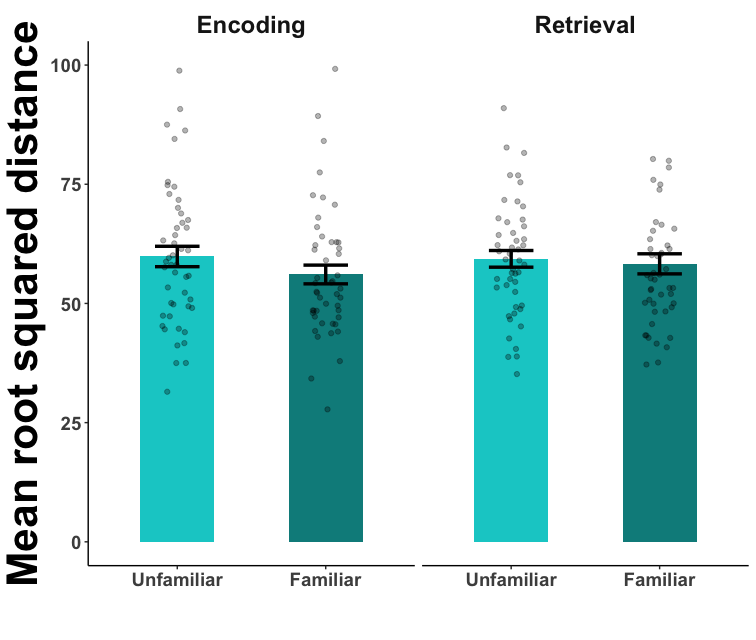


Figure s11. Results of the mean root squared distance analysis in the single-encoding displays.

**Analysis of the mean number of fixations.**

**
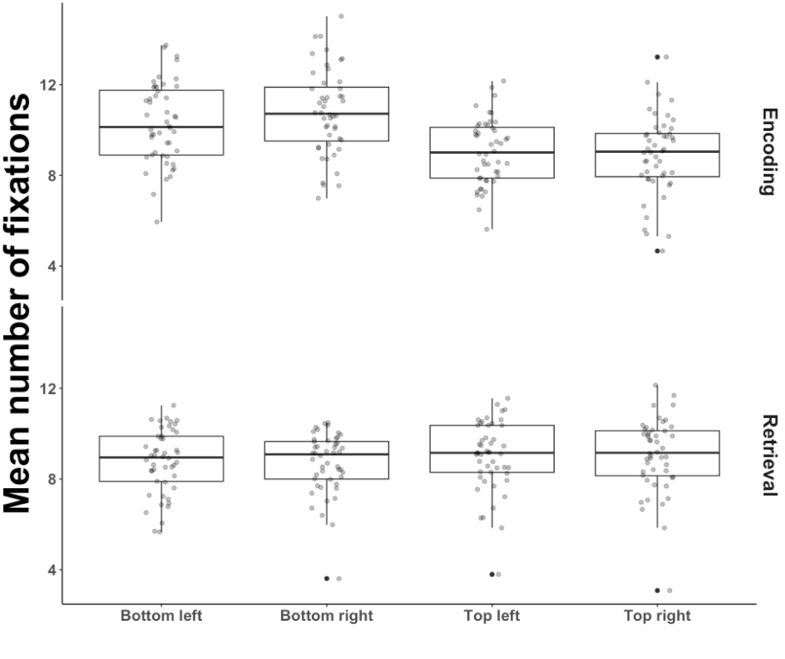
**

Figure S12. The mean number of fixations in the multiple-display across participants. box plots represent the median line, end of boxes show the 25% and 75% borders, and whiskers represents 1.5* interquartile range.

**
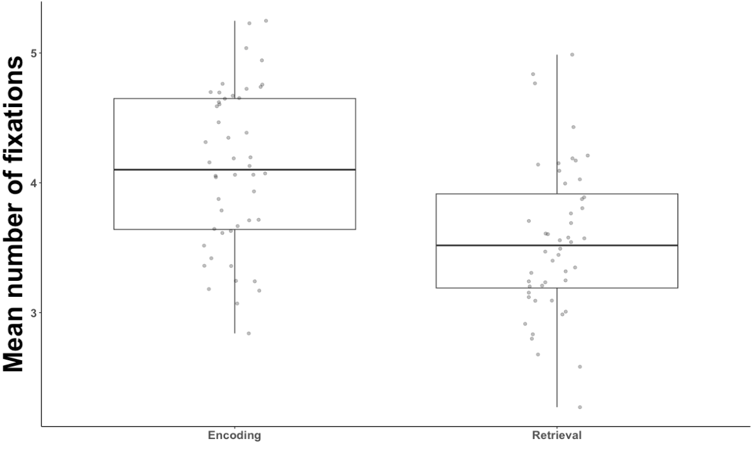
**

Figure S12. The mean number of fixations in the single-display across participants. box plots represent the median line, end of boxes show the 25% and 75% borders, and whiskers represents 1.5* interquartile range.
